# 4-Phenyl-1,3-thiazole-2-amines as scaffolds for new antileishmanial agents

**DOI:** 10.1186/s40409-018-0163-x

**Published:** 2018-09-10

**Authors:** Carina Agostinho Rodrigues, Paloma Freire dos Santos, Marcela Oliveira Legramanti da Costa, Thais Fernanda Amorim Pavani, Patrícia Xander, Mariana Marques Geraldo, Ana Mengarda, Josué de Moraes, Daniela Gonçales Galasse Rando

**Affiliations:** 1 Chemical and Pharmaceutical Research Group, Department of Pharmaceutical Sciences, Institute of Environmental, Chemical and Pharmaceutical Sciences, Federal University of São Paulo (UNIFESP), Rua São Nicolau, 210, 2o andar, Diadema, SP 09913-030 Brazil; 2 Laboratory of Cellular Immunology and Biochemistry of Fungi, Department of Pharmaceutical Sciences, Institute of Environmental, Chemical and Pharmaceutical Sciences, Federal University of São Paulo (UNIFESP), Rua São Nicolau, 210, 2o andar, Diadema, SP 09913-030 Brazil; 3 Research Group of Neglected Diseases, University of Guarulhos, Praça Tereza Cristina, 88, Guarulhos, SP 07020-071 Brazil

**Keywords:** *2*-aminothiazoles, Antikinetoplastids, Antileishmanial, Cutaneous, Target fishing

## Abstract

**Background:**

There is still a need for new alternatives in pharmacological therapy for neglected diseases, as the drugs available show high toxicity and parenteral administration. That is the case for the treatment of leishmaniasis, particularly to the cutaneous clinical form of the disease. In this study, we present the synthesis and biological screening of eight 4-phenyl-1,3-thiazol-2-amines assayed against *Leishmania amazonensis*. Herein we propose that these compounds are good starting points for the search of new antileishmanial drugs by demonstrating some of the structural aspects which could interfere with the observed activity, as well as suggesting potential macromolecular targets.

**Methods:**

The compounds were easily synthesized by the methodology of Hantzsch and Weber, had their purities determined by Gas Chromatography-Mass spectrometry and assayed against the promastigote forms of *Leishmania amazonensis* as well as against two white cell lines (L929 and THP-1) and the monkey’s kidney Vero cells. PrestoBlue® and MTT viability assays were the methodologies applied to measure the antileishmanial and cytotoxic activities, respectively. A molecular modeling target fishing study was performed aiming to propose potential macromolecular targets which could explain the observed biological behavior.

**Results:**

Four out of the eight compounds tested exhibited important anti-promastigote activity associated with good selectivity indexes when considering Vero cells. For the most promising compound, compound **6**, IC_50_ against promastigotes was 20.78 while SI was 5.69. Compounds **3** (IC_50_: 46.63 μM; SI: 26.11) and **4** (IC_50_: 53.12 μM; SI: 4.80) also presented important biological behavior. A target fishing study suggested that S-methyl-5-thioadenosine phosphorylase is a potential target to these compounds, which could be explored to enhance activity and decrease the potential toxic side effects.

**Conclusions:**

This study shows that 4-phenyl-1,3-thiazol-2-amines could be good scaffolds to the development of new antileishmanial agents. The S-methyl-5-thioadenosine phosphorylase could be one of the macromolecular targets involved in the action.

**Electronic supplementary material:**

The online version of this article (10.1186/s40409-018-0163-x) contains supplementary material, which is available to authorized users.

## Background

Neglected diseases affect thousands of people, predominantly in developing countries [1]. The available pharmacological therapies, however, are far from satisfactory, since the existing drugs exhibit a moderate efficacy associated with toxicity. This is the case of leishmaniasis, a tropical endemic disease caused by protozoa of the genus *Leishmania* [2]. The most common leishmaniasis treatments frequently require parenteral administration and chronic therapeutic schemes, which result in high costs and low accessibility to the farthest endemic regions [3]. For these reasons, the need for new alternatives to treat neglected diseases remains urgent.

The 2-aminothiazole scaffold is present in the structure of antiviral [4], antifungal [5], antimicrobial [6], anti-cancerous [7], and anti-inflammatory compounds [8]. Recent papers have reported different 2-aminothiazole derivatives as promising agents against kinetoplastids such as *Trypanosoma cruzi*, *Trypanosoma brucei*, *Leishmania donovani,* and *Leishmania infantum* [9–11].

Kaiser et al. screened a set of 400 drug-like compounds from the chemical library known as “Malaria Box”, made available by the Medicines for Malaria Venture initiative, for potential repurposing of these compounds as antikinetoplastids. Their findings showed that 2-aminothiazole derivatives might be potential hits to be explored against trypanosomatids, specifically against Trypanosomes [9].

More recently, Papadopoulou et al. synthesized a small series of 5-nitro-2-aminothiazoles derivatives which presented better antitrypanosomal activity than the standard drug benznidazole. Antileishmanial activity was also observed in some of the derivatives; however, toxicity against L6 cells was also verified [10].

The 2-aminothiazole ring has gained increasing attention as an antiprotozoal hit. Nevertheless, the 4-phenyl-1,3-thiazole-2-amine scaffold alone has never been explored by its biological potential, mainly regarding the antileishmanial activity.

In this paper, we report the synthesis and biological screening of eight 4-phenyl-1,3-thiazol-2-amines assayed against *L. amazonensis*, a species of leishmania from the *L. mexicana* complex, which is responsible for the cutaneous form of the disease. The compounds were also explored as to assess their cytotoxicity, selectivity index, and potential macromolecular targets.

The main objective of these studies was to understand whether 4-phenyl-1,3-thiazol-2-amines could be good starting points in the search for new antileishmanial drugs, as well as to assess the chemical aspects that could interfere with the activity. Potential macromolecular targets, which could explain the observed actions, were also investigated by target fishing approach.

## Methods

Eight different aromatic ketones were applied in accordance with their 4-phenyl ring substituent, considering their effect on the lipophilicity of the system (Table 1).

**Table 1:** The 4-Phenyl-1,3-thiazol-4-amines series

Compound	R	CLogP^a^	MR^b^	Yield %
01	H	2.61	50.20	61
02	4-CH_3_	3.07	55.25	72
03	4-CH_2_CH_3_	3.47	59.85	59
04	3,4-Cl	3.64	59.81	53
05	4-CF_3_	3.49	56.18	18
06	4-C(CH_3_)_3_	4.23	68.87	27
07	4-OH	2.32	52.19	47
08	4-Cl	3.12	55.01	67

**(a)** Calculated n-Octanol/Water Partition Coefficient; **(b)** Molar Refractivity

### Synthesis

The 4-phenyl-2-aminothiazoles were synthesized according to the methodology of Hantzsch and Weber [12], from their respective arylketones and thiourea (1:2), in the presence of iodine (4 equivalents), by heating the mixture for 4 h. At the end of the reaction, hot water was added to the raw mixture, which was cooled to room temperature and washed with three portions of ethyl ether (~ 10 mL each) to remove the residual iodine. The water phase was neutralized with a saturated solution of ammonium hydroxide and washed with distilled water to yield a solid isolated by filtration under reduced pressure. The pure 4-phenyl-1,3-thiazol-2-amines were obtained by crystallization from Ethanol:water (4:1) solution [13].

### Antileishmanial assay

All compounds were assayed against promastigotes of *Leishmania amazonensis* (MHOM/BR/1973/M2269). Promastigotes were grown in 199 media (Sigma, USA) supplemented with 10% (v/v) of fetal bovine serum, 1% penicillin (10,000 UI/mL)/streptomycin (10.0 mg/mL) (Sigma, USA). The parasites were collected at log phase, they were counted at the Neubauer chamber, their concentration was adjusted to 1 × 10^6^ cell/mL, and their cells were seeded at a 96-wells plate. The 4-phenyl-2-aminothiazoles were added to the culture, considering concentrations ranging from 3.2 to 142 μM. Compounds were diluted in Dimethyl sulfoxide (DMSO) (25 mg/mL) and diluted in 199 media to generate a 1% DMSO starting solution of each test compound. Positive control with no compounds and DMSO control were considered at the assay. All plates were incubated at 25 °C for 48 h, and PrestoBlue® vital dye was added to the wells.

Viability was accessed with PrestoBlue® (Invitrogen, ThermoFisher) according to the manufacturer’s instructions. The reagent was added in each well (10 μL) and plates incubated for 2 h. Then, plates were read on a microplate reader (PowerWave HT, Biotek) with excitation/emission 560/590 nm (nm). The relative fluorescence units were used to generate the quantitative results. All compounds were analyzed as duplicates, and the parameters to calculate IC_50_ were obtained by GraphPad Prism, version 5.0. IC_50_ values were determined by the Hill equation rearranged [14].

$$ {\mathrm{LogIC}}_{50}={\mathrm{logEC}}_{50}-\left\{\log \left(\mathrm{Top}-\mathrm{Bottom}/\mathrm{Y}-\mathrm{Bottom}-1\right)/\mathrm{HillSlope}\right\} $$

### Cytotoxicity assay

The cytotoxicity was measured in L929 and THP1 cell lines using PrestoBlue®Cell Viability Reagent (Thermo Scientific, Invitrogen) as described by Lall et al. with modifications [15]. First, cells were maintained in RPMI 1640 (Gibco) supplemented with 10% fetal bovine serum (FBS) and 1% antibiotics (100 U/mL penicillin, 100 μg/mL streptomycin). Cell cultures were grown and maintained at 37 °C in a humidified incubator with 5% CO_2_. Then, L929 cells were seeded in 96-wells flat microtiter plate at a concentration of 1 × 10^5^cells/mL (100 μL/well). To THP-1 cells, cultures were differentiated into macrophages by adding 50 ng/mL 12-myristate 13-acetate phorbol (PMA) (Calbiochem, San Diego, USA) in RPMI-free FBS. After 24 h of culturing, the adhered cells were washed with PBS and RPMI with 10% FBS were added. Both cell lines were treated with 4-phenyl-2-aminothiazoles at concentrations ranging from 3.2 to 142 μM. The plates were incubated for 48 h and, thereafter, 20 μL/well PrestoBlue® was added. The plates were incubated for further 2 h and the fluorescence was read at bottom using 540–570 nm excitation and 580–610 nm emission. The average of the fluorescence values of the control wells (medium plus PrestoBlue®) was subtracted from the fluorescence value of the positive controls (cells, medium, and PrestoBlue®) and from each experimental well (cells, medium, compounds and PrestoBlue®).

Cytotoxicity was also evaluated using Vero cells, a monkey kidney cell line obtained from the American Type Culture Collection (ATCC CCL-81; Manassas, VA). The cytotoxicity was determined by the 3-(4,5dimethylthiazol- 2-yl)-2,5-diphenyltetrazolium bromide (MTT) method according to a previously described procedure [16, 17].

In all cases, the CC_50_ was calculated as described as in the Antileishmanial assay Section.

### Selectivity index calculation

Selectivity indexes (SI) were calculated by dividing CC_50_ against each mammalian cell line by the IC_50_ against *L. amazonensis* (CC_50_/IC_50_).

### Structure-activity relationship studies

Regarding the studies of the structure-activity relationship, both the partition coefficient and the molar refractivity (CLogP and MR, respectively) were calculated with Marvin Sketch 6.2.2 software [18]. Electronic parameters, such as punctual Mulliken charges and dipole moment values, were calculated to the minimum energy conformer obtained after geometry optimization processing at Gaussian 09 W software. Electrostatic potential charges (ChelpG) were calculated by the ab initio method HF/6-31 g* (Gaussian 09 W) and mapped on the Connolly surface of each molecule [19]. PaDel-descriptor calculator was used to obtain the topological indexes [20]. Only qualitative structure-activity relationship studies were performed, as well as only the linear correlation coefficients above 0.8 were taken into consideration.

### Target fishing and docking studies

Target fishing studies were performed applying the Web-based Software Pharmmapper [21], considering the 3D-optimized structure of the 4-phenyl-1,3-thiazole-2-amines and the ChelpG charges as the input files. The best 300 results were analyzed based on their fit and z’score functions.

The docking protocol was established after performing redocking studies with the crystallographic data retrieved from Protein Data Bank, entry code 1CG6 (resolution: 1.7 Å), using the Gold 5.4.1 software [22]. This protocol was applied to the docking simulation with the test compounds, mainly to the **6** one, the best compound of the series. The binding site was defined by the centroid between Met196 and Asp220 and considering a region of 14 Å around this point. Ten runs with 10 solutions each were performed to generate a set of 100 complexes, which were examined by the incidence of a recurrent pose as well as by their Goldscore and Chemscore values.

## Results

### Synthesis

The compounds were synthesized and isolated as solids of good purity and yields. Structural characterization of these compounds was performed by ^1^H-/^13^C-Nuclear Magnetic Resonance (NMR) and Infrared spectra (IR). Gas Chromatography-Mass Spectrometry (GC-MS) analysis was performed to verify the purity, as well as, the structure of the analogues. All compounds presented purities varying from 98 to 100% and molecular ion values (m/z) and fragmentation patterns correspondent to what was expected for each molecule (Additional file 1).

**Compound 1** was obtained as a pale yellow powder (61%): ^1^H-NMR (300 MHz, [D6] DMSO, ppm): δ=7.00 (s, 1H, H_5_); 7.05 (s, 2H, NH_2_); 7.24 (t, 1H, J_1_ = 6 Hz, J_2_ = 9 Hz, H_13_); 7.35 (t, 2H, J_1_ = 6 Hz, J_2_ = 9 Hz, H_9_ and H_11_); 7.79 (d, 2H, J = 9 Hz, H_8_ and H_12_); ^13^C-NMR (75 MHz, [D6] DMSO, ppm): δ=101.94 (C_5_); 125.99 (C_8,_ and C_12_); 127.62 (C_9_, C_10_ and C_11_); 128.91 (C_4_); 135.40 (C_2_); IR (KBr): 3436 (ν_ass_NH_2_); 3254 (ν_sim_NH_2_); 3155 (νC-H aromatic); 1599 (νC=N), 715 (νC-S-C); GC (retention time, relative intensity in %): 14.10 (100); LRMS (EI, 70 eV) *m/z* calculated for C_9_H_8_N_2_S 176.23, found 176.

**Compound 2** was obtained as a pale yellow powder (72%): ^1^H-NMR (300 MHz, [D6] DMSO, ppm): δ=2.29 (s, 3H, CH_3_); 6.91 (s, 1H, H_5_); 7.01 (s, 2H, NH_2_); 7.16 (d, 2H, J = 6 Hz, H_9_a and H_11_); 7.67 (d, 2H, J = 9 Hz, H_8_ and H_12_); ^13^C-NMR (75 MHz, [D6] DMSO, ppm): δ=21.25 (C_13_); 101.01(C_5_); 125.93 (C_7_); 129.48 (C_8_ and C_12_); 132.76 (C_9_ and C_11_); 136.81 (C_10_); 150.36 (C_4_); 168.53 (C_2_); IR (KBr): 3454 (ν_ass_NH_2_); 3300 (ν_sim_NH_2_); 3117 (νC-H aromatic); 2954 (νC-H CH_3_); 1537 (νC=N), 731 (νC-S-C); GC (retention time, relative intensity in %): 15,46 (100); LRMS (EI, 70 eV) *m/z* calculated for C_10_H_10_N_2_S 190.26, found 190.

**Compound 3** was obtained as a dark yellow powder (59%): ^1^H-NMR (300 MHz, [D6] DMSO, ppm): δ=1.18 (t, 3H, J_1_ = 9 Hz, J_2_ = 6 Hz, CH_3_ ethyl); 2.59 (q, 2H, J_1_ = 9 Hz, J_2_ = 6 Hz, CH_2_ ethyl); 6.91 (s, 1H, H_5_); 7.01 (s, 2H, NH_2_); 7.19 (d, 2H, J = 9 Hz, H_9_ and H_11_); 7.69 (d, 2H, J = 9 Hz, H_8_ and H_12_); ^13^C-NMR (75 MHz, [D6] DMSO, ppm): δ=15.97 (C_14_); 28.35 (C_13_); 101.06 (C_5_); 126.00 (C_7_); 128.27 (C_9_ and C_11_); 133.00 (C_8_ and C_12_); 143.17 (C_10_); 150.36 (C_4_); 168.54 (C_2_); IR (KBr): 3425 (ν_ass_NH_2_); 3265 (ν_sim_NH_2_); 3116 (νC-H aromatic); 2958 (νC-H CH_3_); 2870 (νC-H CH_2_);1516 (νC=N); GC (retention time, relative intensity in %): 16,54 (100); LRMS (EI, 70 eV) MS *m/z* calculated for C_11_H_12_N_2_S 204.29, found 204.

**Compound 4** was obtained as a white powder (53%): ^1^H-NMR (300 MHz, [D6] DMSO, ppm): δ=7.14 (s, 2H, NH_2_); 7.22 (s, 1H, H_5_); 7.61 (d, 1H, J = 9 Hz, H_12_); 7.77 (d, 1H, J = 9 Hz, H_11_); 8.01 (d, 1H, J = 3 Hz, H_8_); ^13^C-NMR (75 MHz, [D6] DMSO, ppm): δ=104.27 (C_5_); 126.00 (C_12_); 127.60 (C_7_); 129.72 (C_8_); 131.17 (C_11_); 131.74 (C_9_ and C_10_); 135.88 (C_4_); 147.65 (C_2_); IR (KBr): 3446 (ν_ass_NH_2_); 3282 (ν_sim_NH_2_); 3124 (νC-H aromatic); 1527 (νC=N); 1022 and 1041(νC-Cl); GC (retention time, relative intensity in %): 18,96 (97,8); LRMS (EI, 70 eV) MS *m/z* calculated for C_9_H_6_Cl_2_N_2_S 245.13, found 244.

**Compound 5** was obtained as a pale yellow powder (18%): ^1^H-NMR (300 MHz, [D6] DMSO, ppm): δ=7.18 (s, 1H, NH_2_); 7.26 (S, 1H, H_5_); 7.73 (d, 2H, J = 9 Hz, H_8_ and H_12_); 8.00 (d, 2H, J = 9 Hz, H_9_ and H _11_); ^13^C-NMR (75 MHz, [D6] DMSO, ppm): δ=104.84 (C_5_); 125.89 (C_9_and C_11_); 126.46 (C_8_ and C_12_); 127.45 (C_10_); 127.98 (C_7_); 138.95 (C_13_); 151.47 (C_4_); 168.96 (C_2_); IR (KBr): 3479 (ν_ass_NH_2_); 3298 (ν_sim_NH_2_); 3143 (νC-H aromatic); 1537 (νC=N); 754 (νC-F); GC (retention time, relative intensity in %): 14,13 (100); LRMS (EI, 70 eV) MS *m/z* calculated for C_10_H_7_F_3_N_2_S 244.24, found 244.

**Compound 6** was obtained as a pale yellow powder (27%): mp: 227-230 °C; ^1^H-NMR (300 MHz, [D6] DMSO, ppm): δ=1.29 (s, 9H, H_14_, H_15_and H_16_); 6.91 (s, 1H, H_5_); 7.02 (s, 2H, NH_2_); 7.37 (d, 2H, J = 9 Hz, H_8_ and H_12_); 7.70 (d, 2H, J = 9 Hz, H_9_ and H_11_). Melting point: Experimental (227-230 °C); Reference (228-230 °C). ^13^C-NMR, IR and GC-MS acquisitions not performed.

**Compound 7** was obtained as a pale yellow powder (47%): ^1^H-NMR (300 MHz, [D6] DMSO, ppm): δ=6.74–6.69 (m, 3H, H_5_, H_8_and H_12_); 6.92 (s, 2H, NH_2_); 7.60–7.55 (m, 2H, H_9_ and H_11_); 9.42 (s, 1H, H_13_); ^13^C-NMR (75 MHz, [D6] DMSO, ppm): δ=98.87 (C_5_); 115.60 (C_9_ and C_11_); 127.35 (C_7_); 150.57 (C_8_ and C_12_); 157.19 (C_10_); 168.41 (C_4_); 206.96 (C_2_); IR (KBr): 3487 (ν_ass_NH_2_); 3377 (ν_sim_NH_2_); 3126 (νC-H aromatic); 2984 (νO-H); 1535 (νC=N); GC (retention time, relative intensity in %): 17,69 (100); LRMS (EI, 70 eV) MS *m/z* calculated for C_9_H_8_N_2_OS 192.24, found 192.

**Compound 8** was obtained as a white powder (67%): ^1^H-NMR (300 MHz, [D6] DMSO, ppm): δ=7.07 (s, 1H, H_5_); 7.08 (s, 2H, NH_2_); 7.39–7.44 (m, 2H, H_8_ and H_9_); 7.83–7.79 (m, 2H, H_9_ and H_11_); ^13^C-NMR (75 MHz, [D6] DMSO, ppm): δ=102.77 (C_5_); 127.69 (C_7_); 128.93 (C_9_ and C_11_); 131.98 (C_8_ and C_12_); 134.23 (C_10_); 143.05 (C_4_); 168.81 (C_2_); IR (KBr): 3437 (ν_ass_NH_2_); 3282 (ν_sim_NH_2_); 3109 (νC-H aromatic); 1533 (νC=N); 826 (νC-Cl); GC (retention time, relative intensity in %): 16,50 (99,6); LRMS (EI, 70 eV) MS *m/z* calculated for C_9_H_7_ClN_2_S 210.68, found 210.

### Biological assays

All compounds were tested against *L. amazonensis* promastigotes. The cytotoxicity was also assessed against L929 and THP-1 by using the PrestoBlue® cell viability reagent, as well as against Vero cell lines by using the MTT viability assay. The results are presented in Table 2.

**Table 2:** IC_50_ values against *L. amazonensis* promastigotes and the cytotoxicity assay results, expressed as CC_50_ values

Compound	IC_50_ (μM)	pIC_50_	CC_50_ (μM) THP1 cells^a)^	SI	CC_50_ (μM) L929 cells^b)^	SI	CC_50_ (μM) VERO cells^c)^	SI
1	957.56	3.02	143.57	0.15	198.26	0.20	710.06	0.74
2	107.68	3.97	135.40	1.26	159.52	1.48	657.51	6.11
3	46.63	4.33	117.27	2.51	95.45	2.05	1217.5	26.11
4	53.12	4.27	84.65	1.59	121.12	2.28	255.03	4.80
5	53.37	4.27	92.21	1.73	106.08	2.00	511.76	9.59
6	20.78	4.68	45.73	2.20	27.07	1.30	118.17	5.69
7	inactive	inactive	inactive	ND	Inactive	ND	651.29	ND
8	115.95	3.94	82.15	0.70	115.06	0.99	595.94	5.14
Amphotericin B	16.23	–	ND	–	ND	–	ND	–
Pentamidine	10.76	–	ND	–	ND	–	ND	–

**(a)** THP1 cells: human monocytic cell line derived from acute monocytic leukemia patients; (**b**) L929 cells: fibroblasts from subcutaneous connective tissue; c) Vero cells: kidney epithelial cells extracted from African green monkeys. IC_50_: Half-maximal inhibitory concentration on promastigotes; pIC_50_: Half-maximal inhibitory concentration in log units; CC_50_: Half cytotoxic concentration; SI: Selectivity Indexes, ND: Not determined. pIC_50_ calculates as pIC_50_ = −log_10_ (IC_50_)

### Structure-activity relationships and molecular modeling studies

Structure-activity relationship analyses revealed a good parabolic correlation between CLogP and antileishmanial activity (Additional file 2A), with a correlation coefficient of 0.968. The same behavior was also observed when regarding the Molar Refractivity (MR) values of the compounds, a parameter which also describes molecular hydrophobicity associated to molecular volume (Additional file 2B).

No significant correlation (r^2^ = > 0.8) was found between activity and electronic parameters, such as electrostatic potential charges, punctual charges or dipole moments (see Additional file 3 to verify the parameters and Additional file 4 for electrostatic potential maps).

However, some relationships could be observed regarding the Electrostatic Potential Maps. Compounds **3** and **6**, for instance, showed a green left-side region (Additional file 4) whereas compounds **4** and **5**, which presented an intermediary activity, exhibited a yellowish left-side region similarly to the one observed with compound **7**, an inactive analogue. These findings could reveal some potential influence of the electronic distribution on the activity, although further studies must be performed to confirm these relationships.

As the cytotoxic effects on Vero cells were substantially different from those obtained against *L. amazonensis*, L-929 and THP-1 cells, a target fishing study was proposed to suggest potential targets and, consequently, mechanisms which could explain these observations.

The web-based software Pharmmapper was applied to search pharmacophore databases, such as the Target Bank, Drug Bank, Binding DB, and Potential Drug Target Database. Such software also takes advantage of a normalized score function, the z’score, to present the most probable targets to compounds with defined chemical structures [21].

The energetically optimized structure of each compound, considering their ChelpG charges, was employed as input, and the best 300 targets – human and non-human – were retrieved. Table 3 lists the five best targets common to these compounds, along with the maximum z’score values that were obtained.

**Table 3:** Pharmmapper results

Target	PDB Code	Ligand Compounds	Max z’score value^a^	Function
S-Methyl-5-thioadenosine phosphorylase	1CG6	All derivatives	3.69	Nucleotide transport and metabolism
Adenosine deaminase	1 V79/1 V89	All but compound **3**	4.72	Nucleotide transport and metabolism
UvrABC System Protein B	1C40	All but compound **4**	3.59	Replication, recombination, and repair
Dihydrofolate reductase	1IA2	All but compound **4**	3.12	Coenzyme transport and metabolism
Queuine t-RNA-ribosyltransferase	1Q66	All but compounds **3** and **4**	3.02	Translation, ribosomal structure and biogenesis

(**a**) among all compounds tested

The human S-Methyl-5-thioadenosine phosphorylase was elected as the best potential target, based in two criteria: the best Max z’score values found to each compound under analysis combined to the fact that this target was returned to all compound submitted to the target fishing.

A BLAST (Basic Local Alignment Search Tool, National Center for Biotechnology Information) search was, then, performed to find homologous proteins in the *L. amazonensis* available proteome [23]. The best nine results are listed in Table 4, together with the statistical analyses which corroborate their significance.

**Table 4:** BLAST results for *Leishmania sp* protein sequences producing significant alignment with human S-methyl-5-thioadenosine phosphorylase

Target	Max Score	Total Score	Query Cover (%)	E value	Identity (%)
Putative methylthioadenosine phosphorylase [*Leishmania infantum* JPCM 5]	182	182	87	2e-55	38
Putative methylthioadenosine phosphorylase [*L. major* strain Friedlin]	180	180	87	1e-54	38
Putative methylthioadenosine phosphorylase [*L. guyanensis*]	182	182	95	3e-55	37
Putative methylthioadenosine phosphorylase [*L. mexicana*MHOM/GT/2001/U1103]	182	182	95	2e-55	37
Putative methylthioadenosine phosphorylase [*L. panamensis*]	179	179	95	2e-54	37
Putative methylthioadenosine phosphorylase [*L. brasiliensis* MHOM/BR/75/M2904]	179	179	95	2e-54	37

**Fig. 1:**
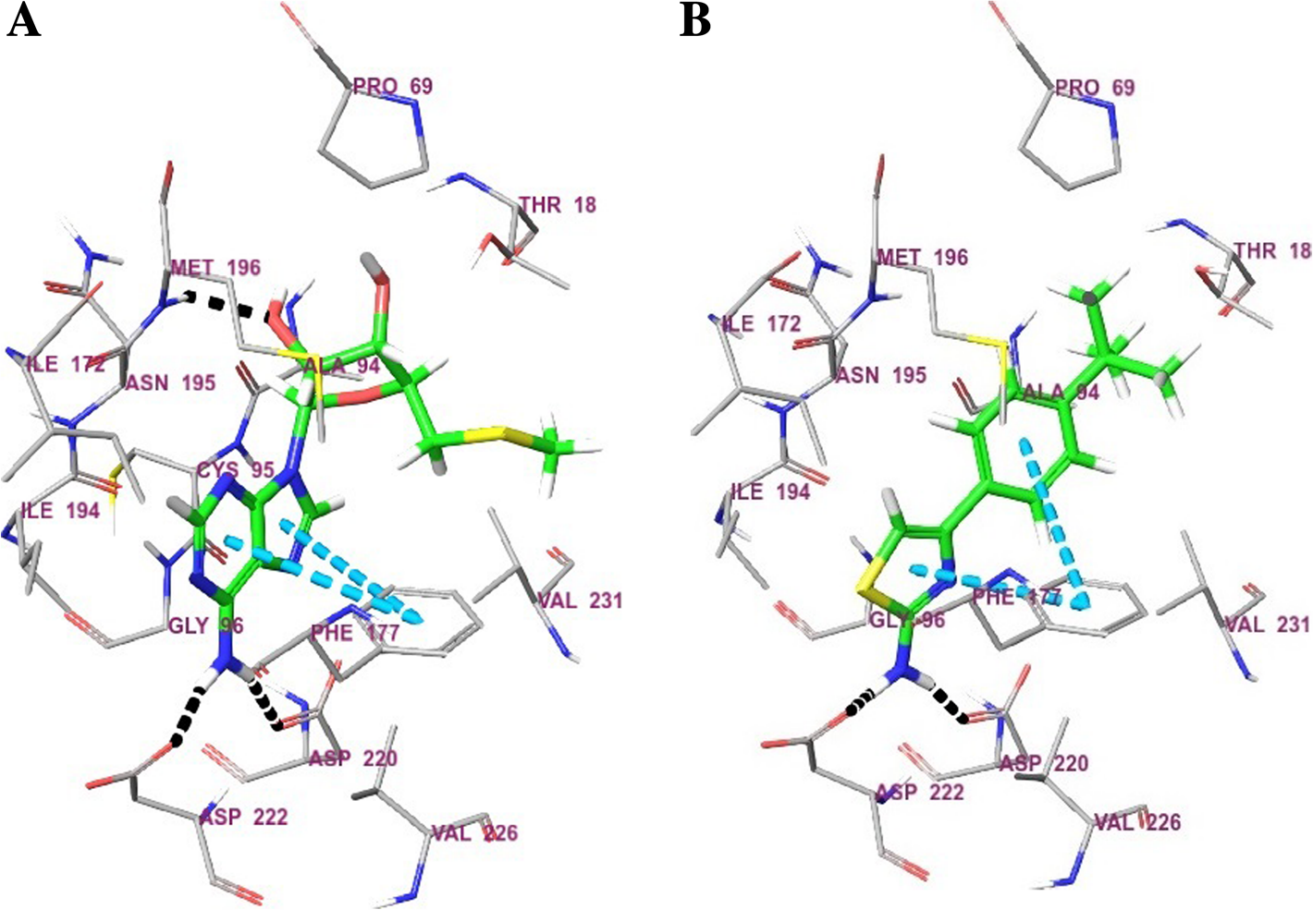
Docking at the S-methyl-5-thioadenosine phosphorylase. **(a)** Co-crystallized ligand. (**b)** Compound **6** docked at the active site. Blue dashed lines: pi-stacking interactions; black dashed lines: hydrogen bonds (For color version, please access the online version of the article)

Identity values above 35% were observed for the *Leishmania* species enzymes, so a docking study considering the human enzyme was also performed to elucidate the potential binding mode of the compounds into the catalytic site. The results are illustrated in Fig. 1.

## Discussions

The compounds were obtained as expected with good yields and purities. The fact of the easy synthetic preparation constitutes one of the major advantages for the future application of these analogues as biological agents, mainly regarding the therapeutics of neglected diseases.

Out of the eight tested analogues, four of them showed activity against the promastigotes of *L. amazonensis* (Table 2), within the range of 20 to 60 μM. Compound **6** was the most active one (21 μM), exhibiting an IC_50_ value close to the one obtained with the standard drug of the assay, the Amphotericin B (16.23 μM). Compound **3** was the second best of the series, followed by Compounds **4** and **5**, both presenting very similar antileishmanial behavior. However, no compound was better than the pentamidine standard drug (10.76 μM).

Even though compounds that were more active against leishmaniasis have been reported in literature, our results are still encouraging because the antileishmanial activity was verified solely considering this scaffold.

A suitable cytotoxic profile was obtained to compound **6** when considering the THP-1 cells, with a selectivity index of 2.20, but this index was lower when considering L929 cells (1.30). The THP-1 cell line is a frequent model for imitating the function and regulation of macrophages, the host cells of *L. amazonensis*.

Similarly, Compounds **3**, **4** and **5** exhibited selectivity indexes suitable to proceed to the next steps of the development, since the toxic dose was about two times higher than the effective dose, both when considering L929 and THP-1 cells. Moreover, no cytotoxic effect was observed against Vero cells at the same concentration range tested, except for compound **1**, which had a SI of 0.74. This last compound was inactive against *L. amazonensis*, though.

These observations encouraged us to proceed with further studies, aiming to understand which of the structural aspects could be related to the activity or cytotoxicity of the compounds.

CLogP values ranging from 3.5 to 4.5 seem to be ideal to the biological activity of the series observed. Moreover, molar refractivity (MR) also presented a good correlation with antileishmanial activity (Additional file 2), indicating that not just the lipophilicity contributes to the antileishmanial activity, but also molar volume and polarizability.

The observed relations with hydrophobic, volume, and polarizability parameters strongly suggest the importance of membrane permeation to the activity of these scaffolds.

Such findings are also in accordance with the observations of Papadopoulou et al., in which linear correlation coefficients of 0.979 and 0.977 were found between the antitrypanosomal activity and the CLogP values of their piperazine and non-piperazine derivatives [10]. Their results against *L. donovani* are significant and have also demonstrated good correlation with the lipophilicity of their analogues (r^2^ = 0.886). These compounds, however, presented cytotoxicity toward L6 cells, which were also related to the lipophilicity.

Aiming to explain the differential cytotoxic profile observed among the eukaryotic cells, molecular modeling studies were also performed.

Initially, a target fishing study was carried out to identify potential targets to the compounds. Most of the returned targets are related to nucleotide recognition, processing, transport, and/or metabolism. This could indicate structural similarity between the scaffold and these endogenous substrates. It also could explain, to some degree, the observed cytotoxicity toward some of the tested cell lines.

The single common target to all tested compounds was the S-methyl-5-thioadenosine phosphorylase (1CG6, pdb entry). This enzyme catalyzes the conversion of 5′-deoxy-5′-methylthioadenosine in adenine and 5-methylthio-D-ribose-1-phosphate in a fundamental step of the polyamine biosynthesis [24]. This pathway is essential to the production of nucleotides and, therefore, essential to cell growth and proliferation.

Unfortunately, the phosphorylase isoform pointed out as a target to these compounds is the human one, and it would not be able to explain the antileishmanial action observed. However, this enzyme has its correspondent isoform in several living organisms.

Investigation for potential *Leishmania sp* isoforms was performed with the BLAST. The search found nine protein sequences with identity values ranging from 37 to 38%, all from different *Leishmania* species, including one from the *L. mexicana* complex. The Expectation values (E-values) of 10^− 54^ obtained in this study corroborates with the significance of the alignment, since the lower the E-value, the more significant the alignment and its scores (Table 4).

**Fig. 2:**
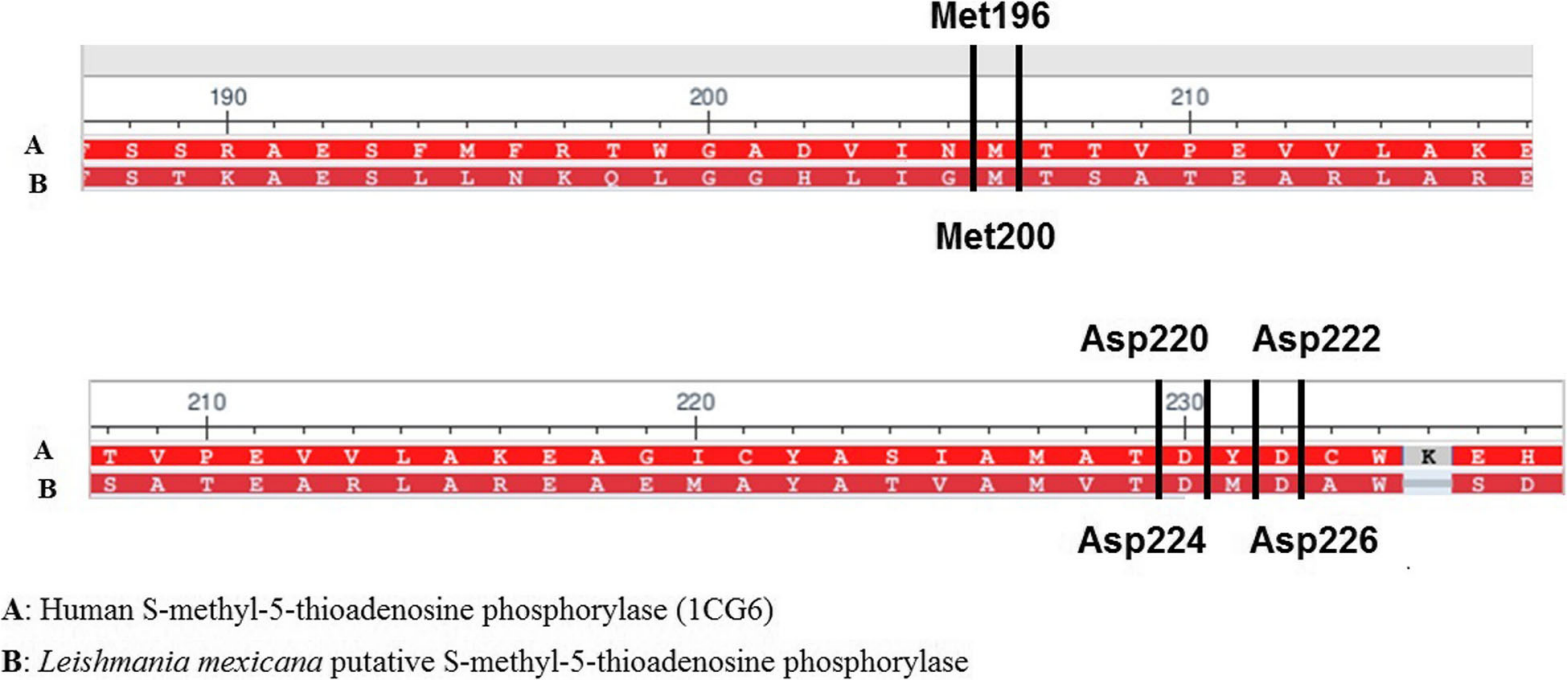
**(a)** Human and (**b**) *L. Mexicana* S-methyl-5-thioadenosine phosphorylase binding site alignment

Most of the conserved residues are located at the binding site region, as revealed by the alignment of the putative *L. mexicana* phosphorylase and the human isoform (Fig. 2).

The binding mode of 5′-deoxy-5′-methylthioadenosine on the human isoform is sustained by three main hydrogen-bound interactions with the Met196, Asp220 and Asp222. The alignment with the enzyme from *L. mexicana* showed that these residues are conserved at the binding site, corresponding to the Met200, Asp224 and Asp226.

This similarity could be the basis for the cytotoxicity observed toward the L929 fibroblasts and THP-1 cells, but not toward the Vero cells, which is a S-methyl-5-thioadenosine phosphorylase negative cell line [25].

In the absence of crystallographic data from the *L. mexicana* enzyme, docking studies with the human isoform were performed to verify the potential binding mode of the 4-phenyl-2-aminothiazole to the phosphorylase (Fig. 1).

The most prevalent pose of compound **6** at the phosphorylase binding site was found at 89% frequency. This pose showed values 43.34Goldscore and 18.93 Chemscore, which are compatible with the good fitness of compound **6** at the binding site.

The docked compound **6** also showed good correspondence with the binding mode exhibited by the 5′-deoxy-5′-methylthioadenosine, the original ligand of the phosphorylase.

The amino group of 4-phenyl-2-aminothiazole compound **6** (Fig. 1b) established hydrogen bonds, as a donor, with the Asp220 and Asp222 residues, just like observed for the co-crystallized ligand (Fig. 1a). Compound **6** also positioned its aromatic systems suitably to establish pi-stacking interactions with Phe117, which, once more, mimics the 5′-deoxy-5′-methylthioadenosine binding mode. The *tert*-butyl group from the 4-phenyl ring was positioned close to a set of hydrophobic residues, Thr18, Pro69 and Val231, occupying the same site of the methylthio moiety from the co-crystallized ligand. Conversely, no interaction between compound **6** and Met196 was observed.

Compounds **3**, **4** and **5** were also theoretically docked to the phosphorylase and exhibited the same pattern of interactions described above when regarding their most frequent poses, 96%, 94% and 100%, respectively (Additional file 5).

Literature on S-methyl-5-thioadenosine phosphorylase expression in different cell lines reports that almost all non-neoplastic mammal cells express this enzyme [26–28]. Most of the S-methyl-5-thioadenosine phosphorylase lacking cells are malignant cells, as discussed by Kamatani et al. [29].

Even though these studies were performed taking the human enzyme into consideration, the results could be used as a guide to future design new antileishmanial compounds. For instance, homology modeling studies can be useful in order to improve such studies.

Derivatives of 2-aminothiazoles have been reported as predominantly active against trypanosomes, such as in the case of Kaiser et al., but they have not shown expressive activity against leishmanias. Nearly all literature reports are based on studies with *L. donovani* or *L. infantum*, both responsible for the visceral leishmaniasis, since this is the lethal form of the disease [9].

Unfortunately, cutaneous leishmaniasis is thought to be less dangerous, and because of it, it is believed to potentially lead to a spontaneous cure. Several cases, however, evolve to the mucocutaneous form of the diseases, just like observed in infections caused by the *L. braziliensis*. This clinical manifestation is characterized by deforming lesions that often compromise the patient’s social life.

Moreover, leishmania species from the *L. mexicana* complex are widely known to be resistant to treatment, mainly those based on in situ free-radical production, as is the case of nitroderivatives. Such mechanism of action implies low selectivity and, consequently, a broad panel of side effects. Ironically, several of the promising new compounds explored against trypanosomatidae parasites are nitroaromatic compounds [30, 31].

Finding compounds that are active against such parasites with IC_50_ values lower than 10 μM and with good toxicological profile is still a challenging task. From this point of view, the 4-phenyl-2-aminothiazole scaffold arises as a non-nitroaromatic alternative to the development of new antileishmanial drugs.

## Conclusion

Considering the results herein reported, the 4-phenyl-2-aminothiazoles might be potential scaffolds to be explored in the search for new antileishmanial hits. Four out of the eight tested compounds exhibited important anti-promastigote activity associated with good selectivity indexes, except in the cases of compounds **1** and **6** (in the latter, only when considering its activity against L929 cells).

This is the first report of their effectiveness against the *L. amazonensis*, one of the species of leishmania responsible for the cutaneous form of the disease. Currently, studies using the most promising compounds have been conducted to evaluate their effects on the amastigote form of the parasite, which is found in the mammalian host. Such tests are important and fundamental for a better evaluation of the applicability of the compounds for the treatment of leishmaniasis.

The 4-phenyl-2-aminothiazole scaffold arises, as discussed, as a non-nitroaromatic alternative to the development of new antileishmanial drugs. As scaffolds, these structures could be rationally modified to present selectivity along with improved antileishmanial activity, since the differences between the binding sites of the human and leishmania phosphorylases can be explored.

## Additional files

Additional file 1: GC-MS results, under the following conditions: Inlet temperature: 250 °C; Oven: initial temperature 80 °C, 10 °C/min up to 250 °C, kept for 13 min; Column RTX-5MS (30 m × 0.25 mm × 0.25 μm). (DOCX 342 kb)

Additional file 2: Structure-Activity Relationships. (**a**) CLogP versus Biological Activity Graphic. Parabolic Correlation coefficient, r^2^ = 0.968. (**b**) MR versus Biological Activity Graphic. Parabolic Correlation coefficient, r^2^ = 0.945. (DOCX 40 kb)

Additional file 3: Physical chemical parameters calculated. (DOCX 14 kb)

Additional file 4: Electrostatic potential maps. (DOCX 2496 kb)

Additional file 5: GPQF-03, GPQF-04, and GPQF-05 best docking poses at S-methyl-5-thioadenosine phosphorylase (1CG6). (DOCX 284 kb)

## References

[1] World Health Organization – Leishmaniasis. http://www.who.int/leishmaniasis/en/. Accessed 24 Jan 2016.

[2] World Health Organization. Control of the leishmaniasis: report of a meeting of the WHO Expert Committee on the Control of Leishmaniases, Geneva; 2010. p. 55–65.

[3] Croft SL (2005). Public-private partnership: from there to here. Trans R Soc Trop Med Hyg.

[4] Ghaemmaghami S, May BCH, Renslo AR, Prusiner SB (2010). Discovery of 2-aminothiazoles as potent antiprion compounds. J Virol.

[5] Amnerkar ND, Bhusari KP (2011). Synthesis of some thiazolyl aminobenzothiazole derivatives as potential antibacterial, antifungal and anthelmintic agents. J Enzyme Inhib Med Chem.

[6] Khan KM, Ambreen N, Karim A, Saied S, Amyn A, Ahmed A (2012). Schiff bases of thiazole as antibacterial and antifungal agents. J Pharm Res.

[7] Gorczynski MJ, Leal RM, Mooberry SL, Bushweller JH, Brown ML (2004). Synthesis and evaluation of substituted 4-aryloxy- and 4-arylsulfanyl-phenyl-2-aminothiazoles as inhibitors of human breast cancer cell proliferation. Bioorg Med Chem.

[8] Karabasanagouda T, Vasudeva A (2008). Synthesis of some new 2-(4-alkylthiophenoxy)-4-substituded-1,3-thiazoles as possible anti-inflammatory and antimicrobial agents. Indian J Chem.

[9] Kaiser M, Maes L, Tadoori LP, Spangenberg T, Ioset JR (2015). Repurposing of the open access malaria box for kinetoplastid diseases identifies novel active scaffolds against trypanosomatids. J Biomol Screen.

[10] Papadopoulou MV, Bloomer WD, Rosenzweig HS, Wilkinson SR, Szular J, Kaiser M (2016). Antitrypanosomal activity of 5-nitro-2-aminothiazole-based compounds. Eur J Med Chem.

[11] Bilbao-Ramos P, Galiana-Roselló C, Dea-Ayuela MA, González-Alvarez M, Vega C, Rolón M (2012). Nuclease activity and ultrastructural effects of new sulfonamides with anti-leishmanial and trypanocidal activities. Parasitol Int.

[12] Hantzsch A, Weber JH (1887). Ueber Verbindungen des Thiasola (Pyridine der Thiophenreihe). Dtsch Chem Gesellschaft.

[13] Carroll King L, Hlavacek RJ (1950). The reaction of ketones with iodine and thiourea. J Am Chem Soc.

[14] Directorate E, Meeting J, Committee C, Working THE, On P, The OF (2010). Guidance document on using cytotoxicity tests to estimate starting doses for acute oral systemic toxicity tests. Organ Econ Co-operation Dev [Internet].

[15] Lall N, Henley-Smith CJ, De Canha MN, Oosthuizen CB, Berrington D (2013). Viability reagent, prestoblue, in comparison with other available reagents, utilized in cytotoxicity and antimicrobial assays. Int J Microbiol.

[16] de Moraes J, Dario BS, Couto RAA, Pinto PLS, da Costa Ferreira AM (2015). Antischistosomal activity of oxindolimine-metal complexes. Antimicrob Agents Chemother.

[17] de Brito MRM, Peláez WJ, Faillace MS, Militão GCG, Almeida JRGS, Argüello GA (2017). Cyclohexene-fused 1,3-oxazines with selective antibacterial and antiparasitic action and low cytotoxic effects. Toxicol in Vitro.

[18] MarvinSketch (version 6.2.2), calculation module developed by ChemAxon. 2014. http://www.chemaxon.com/products/marvin/marvinsketch/.

[19] Frisch MJ, Trucks GW, Schlegel HB, Scuseria GE, Robb MA, Cheeseman JR (2009). Gaussian 09. Revision D.1.

[20] Yap CW (2011). PaDEL-descriptor: an open source software to calculate molecular descriptors and fingerprints. J Comput Chem.

[21] Liu X, Ouyang S, Yu B, Liu Y, Huang K, Gong J (2010). PharmMapper server: a web server for potential drug target identification using pharmacophore mapping approach. Nucleic Acids Res.

[22] Jones G, Willett P, Glen RC, Leach AR, Taylor R (1997). Development and validation of a genetic algorithm for flexible docking. J Mol Biol.

[23] Altschul SF, Gish W, Miller W, Myers EW, Lipman DJ (1990). Basic local alignment search tool. J Mol Biol.

[24] Appleby TC, Erion MD, Ealick SE (1999). The structure of human 5′-deoxy-5′-methylthioadenosine phosphorylase at 1.7 a resolution provides insights into substrate binding and catalysis. Structure.

[25] Della Ragione F, Russo G, Oliva A, Mastropietro S, Mancini A, Borrelli A (1995). 5′-Deoxy-5′-methylthioadenosine phosphorylase and p16INK4 deficiency in multiple tumor cell lines. Oncogene.

[26] Williams-Ashman HG, Seidenfeld J, Galletti P (1982). Trends in the biochemical pharmacology of 5′-deoxy-5′-methylthioadenosine. Biochem Pharmacol.

[27] Carrera CJ, Eddy RL, Shows TB, Carson DA (1984). Assignment of the gene for methylthioadenosine phosphorylase to human chromosome 9 by mouse-human somatic cell hybridization. Proc Natl Acad Sci U S A.

[28] Della Ragione F, Oliva A, Palumbo R, Russo GL, Gragnaniello V, Zappia V (1992). Deficiency of 5′-deoxy-5′-methylthioadenosine phosphorylase activity in malignancy. Absence of the protein in human enzyme-deficient cell lines. Biochem J.

[29] Kamatani N, Nelson-Rees WA, Carson DA (1981). Selective killing of human malignant cell lines deficient in methylthioadenosine phosphorylase, a purine metabolic enzyme. Proc Natl Acad Sci U S A.

[30] Rando DG, Avery MA, Tekwani BL, Khan SI, Ferreira EI (2008). Antileishmanial activity screening of 5-nitro-2-heterocyclic benzylidene hydrazides. Bioorg Med Chem.

[31] Soong L, Henard CA, Melby PC (2012). Immunopathogenesis of non-healing American cutaneous leishmaniasis and progressive visceral leishmaniasis. Semin Immunopathol.

