# Supplementary material for: 4-Phenyl-1,3-thiazole-2-amines as scaffolds for new antileishmanial agents
Source: J Venom Anim Toxins Incl Trop Dis. 2018 Sep 10;24:26. doi: 10.1186/s40409-018-0163-x (PMC6131760; doi:10.1186/s40409-018-0163-x)
Supplement: Supplementary file 1 — GC-MS results, under the following conditions: Inlet temperature: 250 °C; Oven: initial temperature 80 °C, 10 °C/min up to 250 °C, kept for 13 min; Column RTX-5MS (30 m × 0.25 mm × 0.25 μm). (DOCX 342 kb) [file 40409_2018_163_MOESM1_ESM.docx]

**Additional file 1**

| **GPQF-01**  **Gas chromatography**  **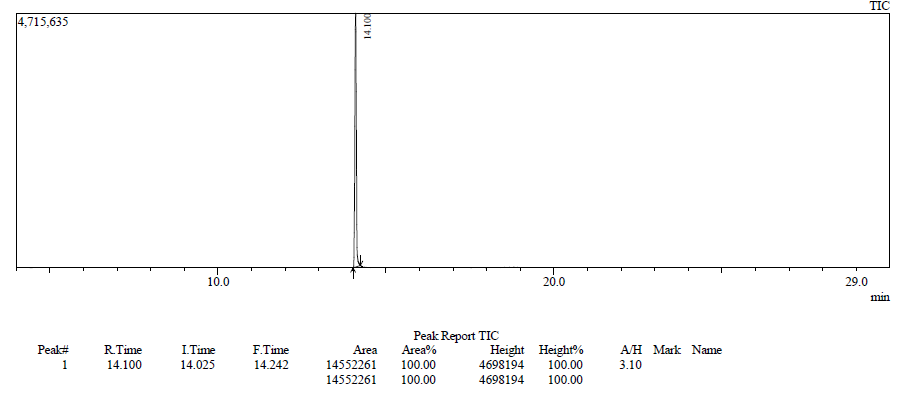**  **Mass Spectrum**  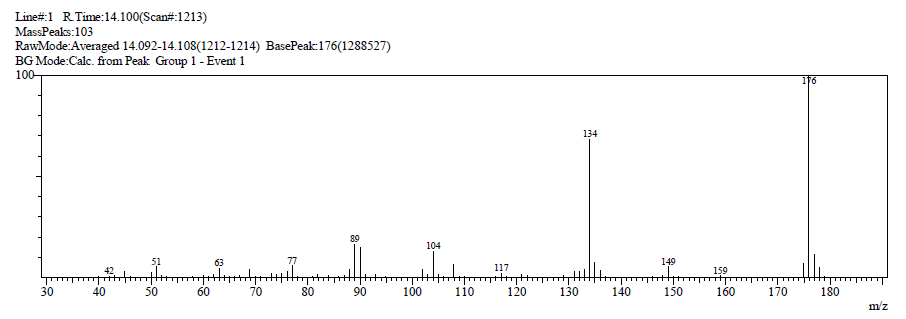  MS m/z calcd for C_9_H_8_N_2_S 176.23, found 176 |
| --- |
| **GPQF-02**  **Gas chromatography**  **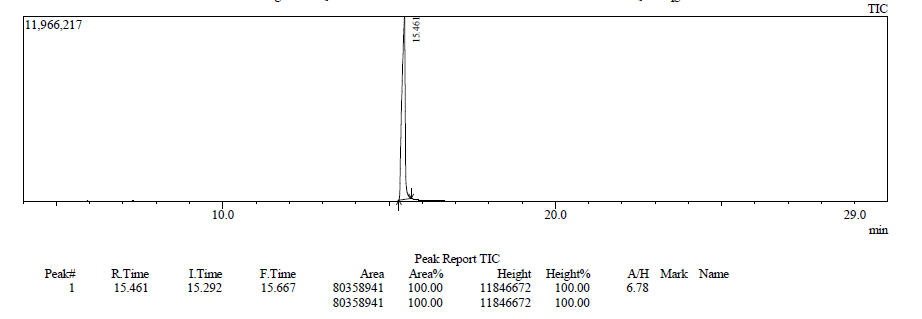**  **Mass Spectrum**  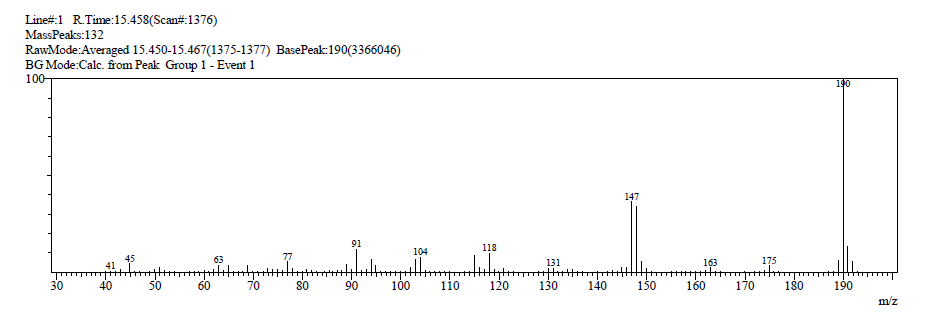  MS m/z calcd for C_10_H_10_N_2_S 190.26, found 190 |
| **GPQF-03**  **Gas chromatography**  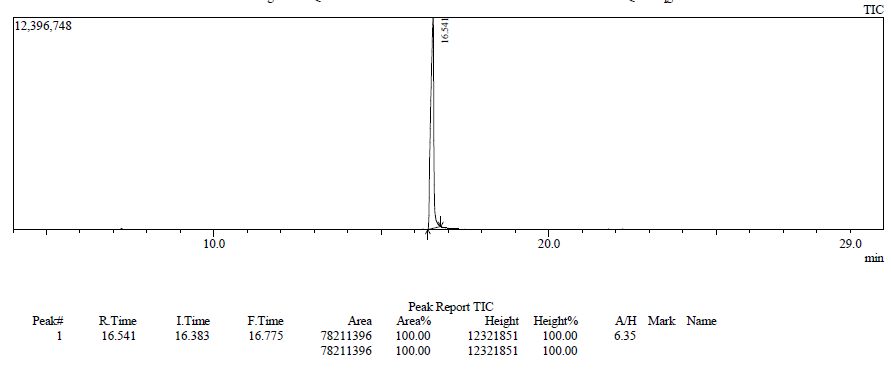  **Mass Spectrum**  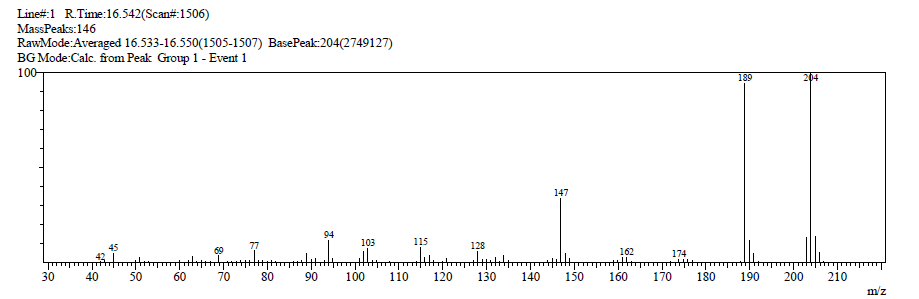  MS m/z calcd for C_11_H_12_N_2_S 204.29, found 204 |
| **GPQF-04**  **Gas chromatography**  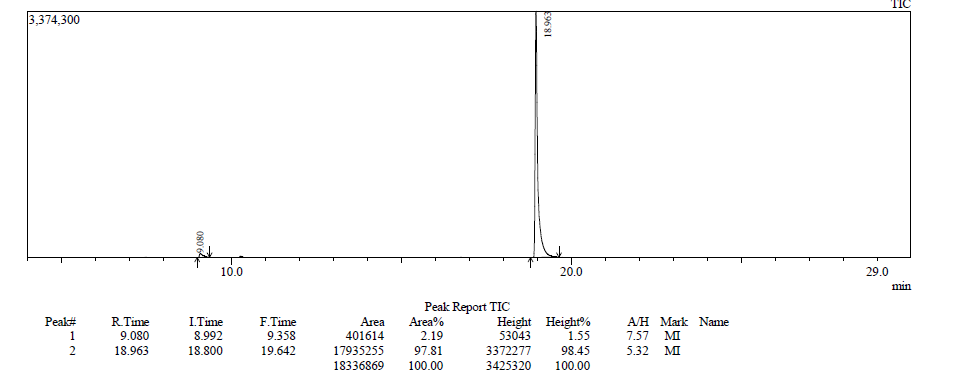  **Mass Spectrum**  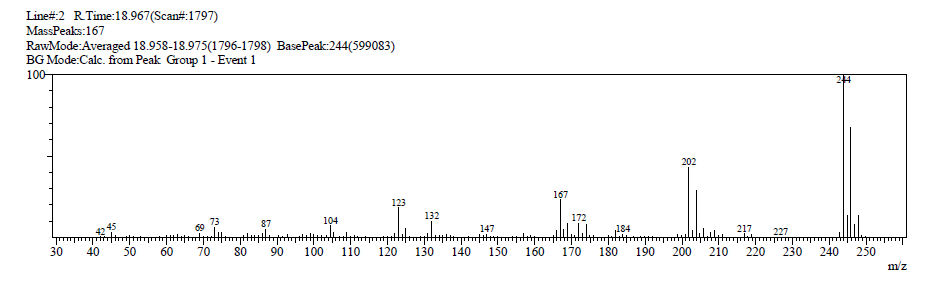  MS m/z calcd for C_9_H_6_Cl_2_N_2_S 245.13, found 244 |
| **GPQF-05**  **Gas chromatography**  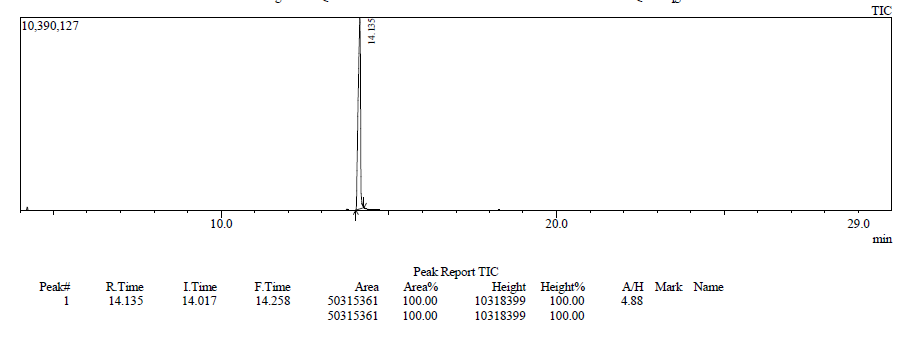  **Mass Spectrum**  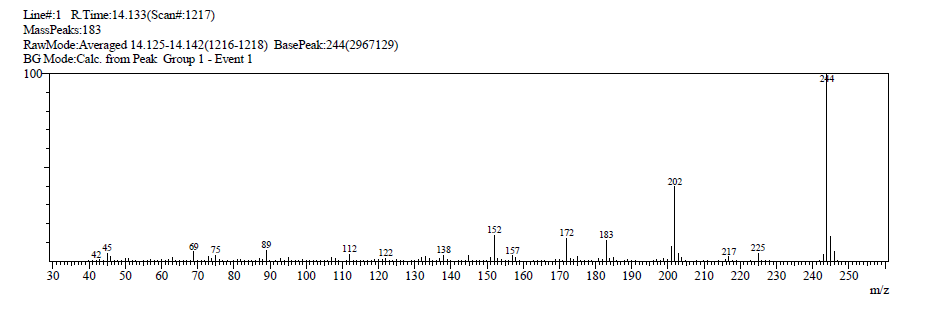  MS m/z calcd for C_10_H_7_F_3_N_2_S 244.24, found 244 |
| **GPQF-07**  **Gas chromatography**  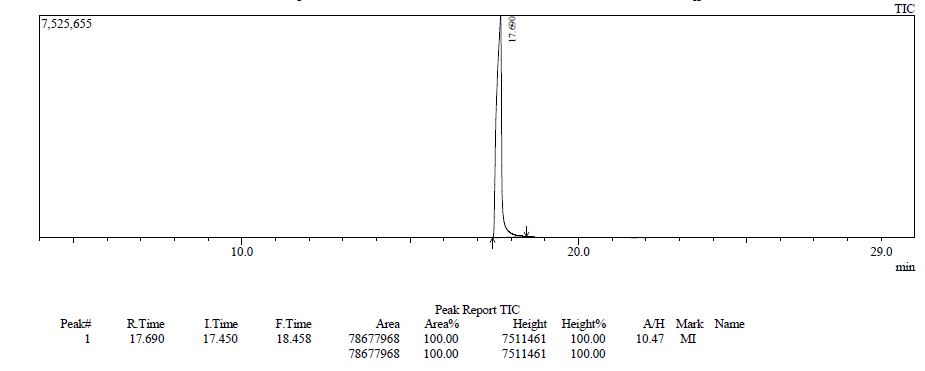  **Mass Spectrum**  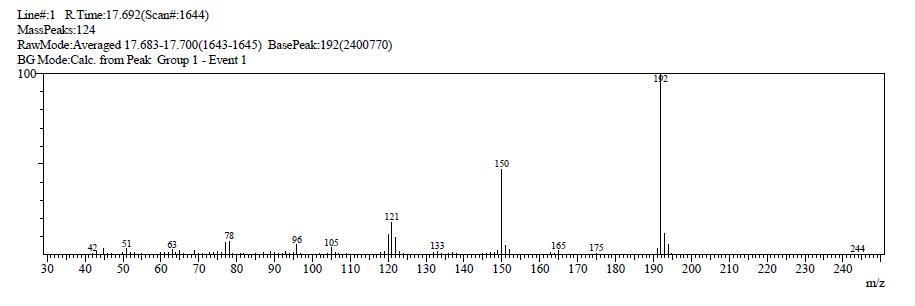  MS m/z calcd for C_9_H_8_N_2_OS 192.24, found 192 |
| **GPQF-08**  **Gas chromatography**  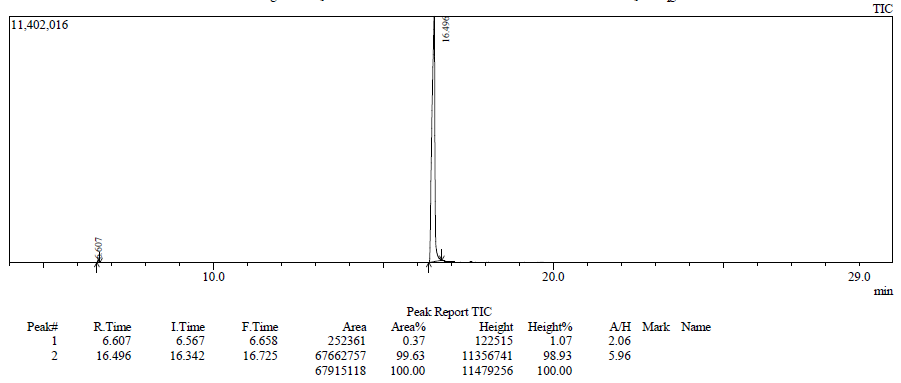  **Mass Spectrum**  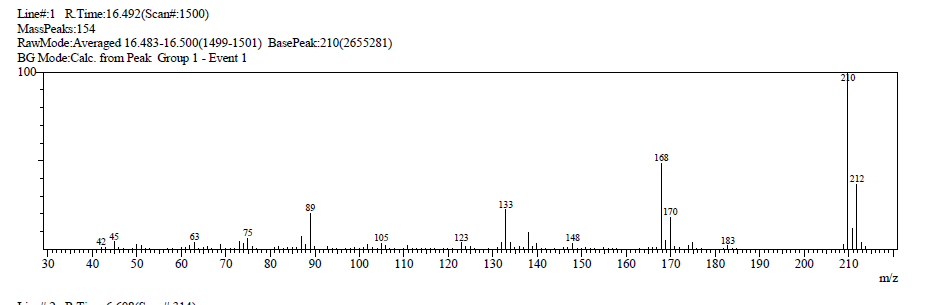  MS m/z calcd for C_9_H_7_ClN_2_S 210.68, found 210 |

NOTE: Compound GPQF-06 was not analyzed by GC-MS because it was obtained in amounts only sufficient to perform some of the structural characterizations and the biological assays. Its purity was verified, then, by melting point determination: Experimental: 227-230 ^o^C; Reference: 228-230 ^o^C.
