# Supplementary figures and images for: 4-Phenyl-1,3-thiazole-2-amines as scaffolds for new antileishmanial agents
Source: J Venom Anim Toxins Incl Trop Dis. 2018 Sep 10;24:26. doi: 10.1186/s40409-018-0163-x (PMC6131760; doi:10.1186/s40409-018-0163-x)

**Additional file 2**


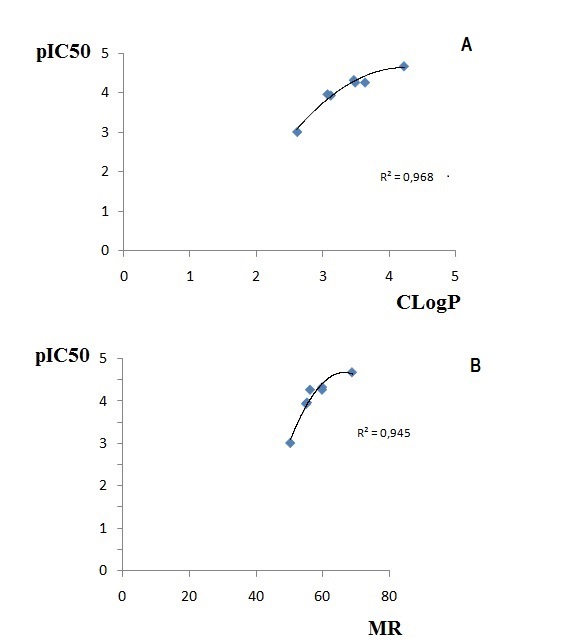

Supplement: Supplementary file 2 — Structure-Activity Relationships. (a) CLogP versus Biological Activity Graphic. Parabolic Correlation coefficient, r2 = 0.968. (b) MR versus Biological Activity Graphic. Parabolic Correlation coefficient, r2 = 0.945. (DOCX 40 kb) [file 40409_2018_163_MOESM2_ESM.docx]

**Additional file 4.** Electrostatic potential maps

| 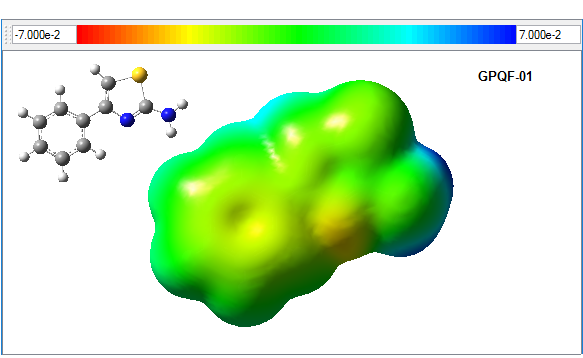 | 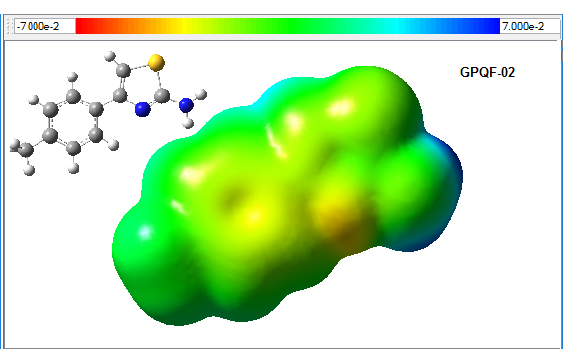 |
| --- | --- |
| 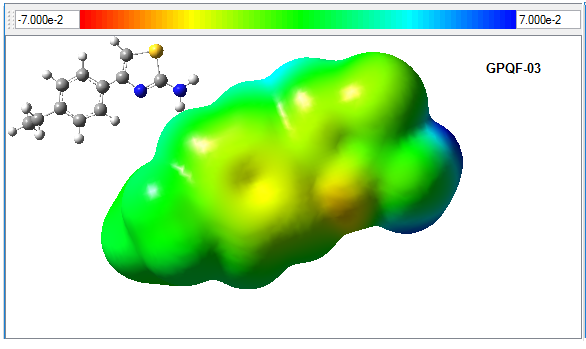 | 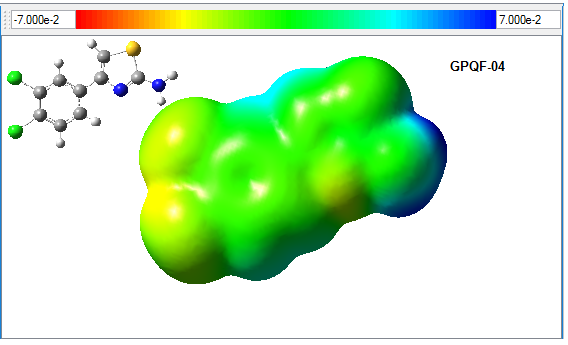 |
| 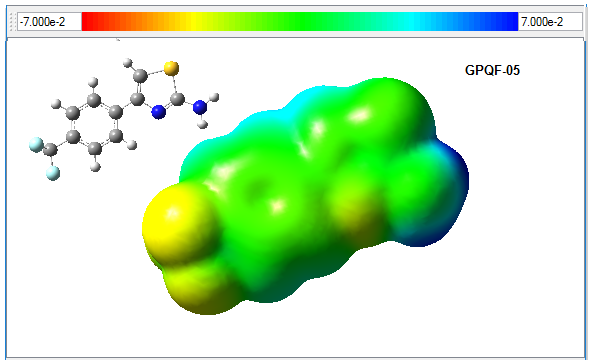 | 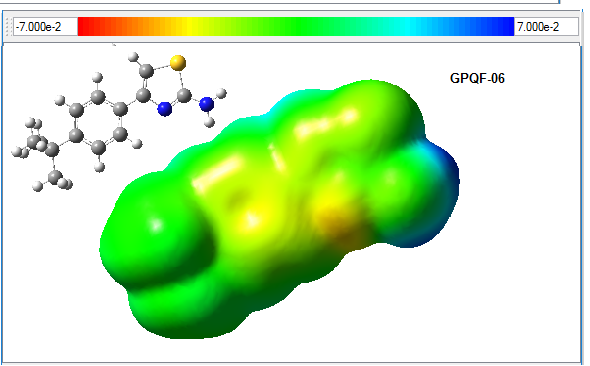 |
| 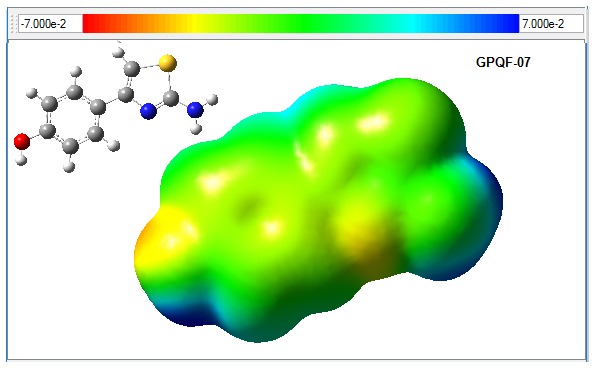 | 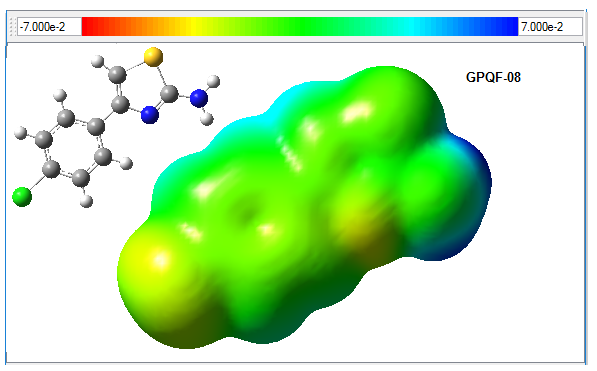 |

Supplement: Supplementary file 4 — Electrostatic potential maps. (DOCX 2496 kb) [file 40409_2018_163_MOESM4_ESM.docx]

**Additional file 5.**

| 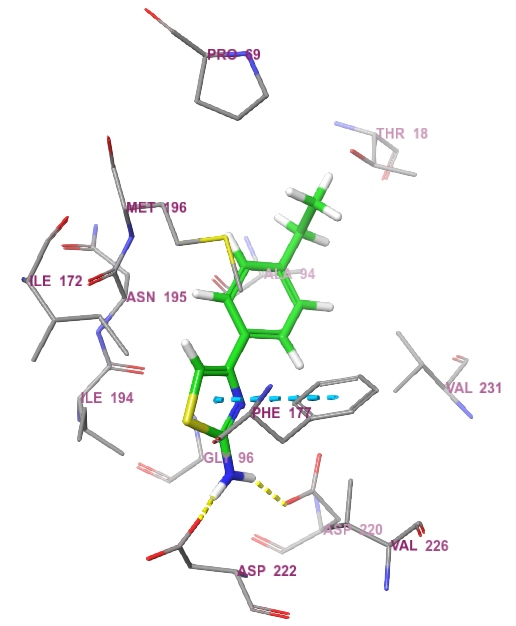  GPQF-03 |
| --- |
| 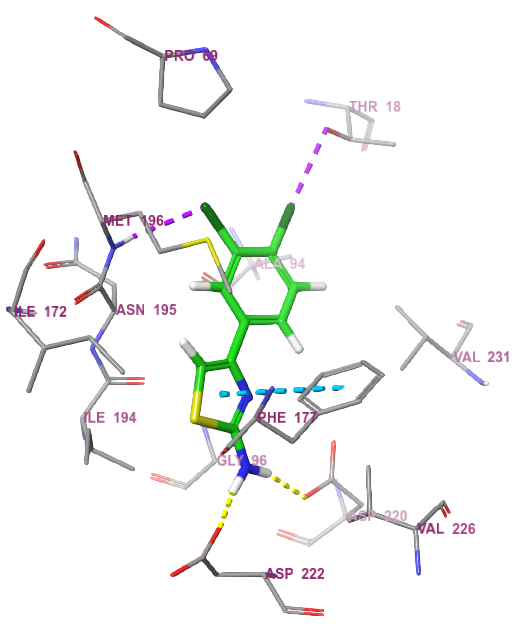  GPQF-04 |
| 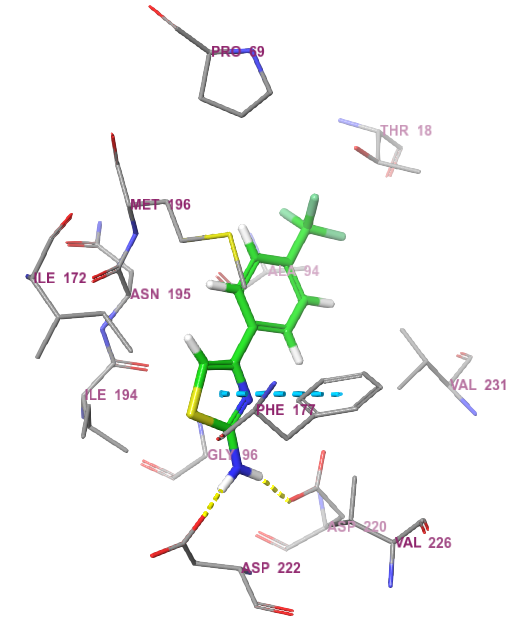  GPQF-05 |

Supplement: Supplementary file 5 — GPQF-03, GPQF-04, and GPQF-05 best docking poses at S-methyl-5-thioadenosine phosphorylase (1CG6). (DOCX 284 kb) [file 40409_2018_163_MOESM5_ESM.docx]
