# Supplementary material for: 4-Phenyl-1,3-thiazole-2-amines as scaffolds for new antileishmanial agents
Source: J Venom Anim Toxins Incl Trop Dis. 2018 Sep 10;24:26. doi: 10.1186/s40409-018-0163-x (PMC6131760; doi:10.1186/s40409-018-0163-x)
Supplement: Supplementary file 3 — Physical chemical parameters calculated. (DOCX 14 kb) [file 40409_2018_163_MOESM3_ESM.docx]

**Additional file 3**

| **GPQF** | **1** | **2** | **3** | **4** | **5** | **6** | **7** | **8** | **r2*** |
| --- | --- | --- | --- | --- | --- | --- | --- | --- | --- |
| **pIC_50_** | 3.02 | 3.97 | 4.33 | 4.27 | 4.27 | 4.68 |  | 3.94 | - |
| **Polarizability** | 20.19 | 21.96 | 23.26 | 23.59 | 21.39 | 26.92 | 19.8 | 21.32 | 0.630 |
| **Platt Index** | 64 | 76 | 88 | 64 | 76 | 112 | 66 | 64 | 0.444 |
| **Randic index** | 9.25 | 10.46 | 11.71 | 9.25 | 10.46 | 14.21 | 9.79 | 9.25 | 0.443 |
| **Balaban index** | 1.68 | 1.55 | 1.78 | 1.87 | 1.6 | 1.6 | 1.55 | 1.55 | 0.005 |
| **Harary index** | 30.74 | 34.6 | 38.28 | 38.98 | 47.14 | 47.14 | 34.6 | 34.6 | 0.654 |
| **Szeged Index** | 303 | 387 | 484 | 463 | 684 | 684 | 387 | 387 | 0.629 |
| **Wierner Index** | 197 | 251 | 318 | 302 | 458 | 458 | 251 | 251 | 0.613 |
| **Wierner Polarity** | 13 | 15 | 17 | 18 | 21 | 21 | 15 | 15 | 0.700 |
| **MDX** | 1.112 | -0.248 | 1.182 | -2.638 | -0.015 | -0.765 | -1.596 | -2.075 | 0.116 |
| **MDY** | 0.895 | -0.867 | 0.756 | 5.191 | 6.159 | 0.485 | -2.351 | -3.771 | 0.050 |
| **MDZ** | 0 | 0 | 0.1288 | 0 | 0 | 0 | 0 | 0 | 0.048 |
| **MDtot** | 1.427 | 0.902 | 1.409 | 5.823 | 6.159 | 0.906 | 2.842 | 4.305 | 0.026 |
| **Char N-CN** | -0.688 | -0.725 | -0.743 | -0.712 | -0.720 | -0.725 | -0.737 | -0.706 | 0.614 |
| **Char C-NH** | 0.932 | 0.955 | 0.876 | 0.921 | 0.968 | 0.965 | 0.935 | 0.957 | 0.002 |
| **Char N-H_2_** | -1.237 | -1.260 | -1.172 | -1.235 | -1.284 | -1.278 | -1.237 | -1.277 | 0.007 |
| **Ehomo** | -0.295 | -0.288 | -0.271 | -0.313 | -0.315 | -0.288 | -0.288 | -0.304 | 0.003 |
| **Elumo** | 0.060 | 0.061 | 0.059 | 0.052 | 0.053 | 0.062 | 0.059 | 0.055 | 0.025 |
| **Polar Surface**  **Area** | 38.91 | 38.91 | 38.91 | 38.91 | 38.91 | 38.91 | 59.14 | 38.91 | 2.0x10^-13^ |

Marvin Beans: Polarizability, Platt Index, Randic Index, Balaban index, Harary index, Szeged Index, Wierner Index, Wierner Polarity, Polar Surface Area

Gaussian 09: MDX= dipole moment x-axis,MDY= dipole moment y-axis, MDZ= dipole moment z-axis, MDtot=total dipole moment, Ehomo=HOMO energy. Elumo=LUMO energy and the Charges at different atoms: Char N-CN= charge of the nitrogen atom from the thiazole ring, Char C-NH= charge of the carbon bonded to the amino group at position 2 of the thiazole ring, Char N-H_2_= charge of the nitrogen of the amino group.

* r^2^ = correlation coefficient between pIC_50_ and the property described at each row.
